# Microbiota modulates the steroid response to acute immune stress in male mice

**DOI:** 10.3389/fimmu.2024.1330094

**Published:** 2024-02-01

**Authors:** Karla Vagnerová, Taťána Gazárková, Martin Vodička, Peter Ergang, Petra Klusoňová, Tomáš Hudcovic, Dagmar Šrůtková, Petra Petr Hermanová, Lucie Nováková, Jiří Pácha

**Affiliations:** ^1^ Institute of Physiology, Czech Academy of Sciences, Prague, Czechia; ^2^ Department of Analytical Chemistry, Faculty of Pharmacy, Charles University, Hradec Králové, Czechia; ^3^ Institute of Microbiology, Czech Academy of Sciences, Nový Hrádek, Czechia; ^4^ Department of Physiology, Faculty of Science, Charles University, Prague, Czechia

**Keywords:** immune stress, gut microbiota, germ-free, mice, anti-CD3, steroids, steroidogenic genes

## Abstract

Microbiota plays a role in shaping the HPA-axis response to psychological stressors. To examine the role of microbiota in response to acute immune stressor, we stimulated the adaptive immune system by anti-CD3 antibody injection and investigated the expression of adrenal steroidogenic enzymes and profiling of plasma corticosteroids and their metabolites in specific pathogen-free (SPF) and germ-free (GF) mice. Using UHPLC-MS/MS, we showed that 4 hours after immune challenge the plasma levels of pregnenolone, progesterone, 11-deoxycorticosterone, corticosterone (CORT), 11-dehydroCORT and their 3α/β-, 5α-, and 20α-reduced metabolites were increased in SPF mice, but in their GF counterparts, only CORT was increased. Neither immune stress nor microbiota changed the mRNA and protein levels of enzymes of adrenal steroidogenesis. In contrast, immune stress resulted in downregulated expression of steroidogenic genes (*Star*, *Cyp11a1*, *Hsd3b1*, *Hsd3b6*) and upregulated expression of genes of the 3α-hydroxysteroid oxidoreductase pathway (*Akr1c21*, *Dhrs9*) in the testes of SPF mice. In the liver, immune stress downregulated the expression of genes encoding enzymes with 3β-hydroxysteroid dehydrogenase (HSD) (*Hsd3b2*, *Hsd3b3*, *Hsd3b4*, *Hsd3b5*), 3α-HSD (*Akr1c14*), 20α-HSD (*Akr1c6*, *Hsd17b1*, *Hsd17b2*) and 5α-reductase (*Srd5a1*) activities, except for *Dhrs9*, which was upregulated. In the colon, microbiota downregulated *Cyp11a1* and modulated the response of *Hsd11b1* and *Hsd11b2* expression to immune stress. These data underline the role of microbiota in shaping the response to immune stressor. Microbiota modulates the stress-induced increase in C_21_ steroids, including those that are neuroactive that could play a role in alteration of HPA axis response to stress in GF animals.

## Introduction

To maintain body homeostasis and protect the survival of individuals from stressful physical and psychological challenges, vertebrates have evolved complex and conserved mechanisms known as stress response (1). In response to stressors, the sympatho-adrenomedullary system and hypothalamic-pituitary-adrenal (HPA) axes are activated. The HPA axis is activated primarily by hypothalamic hormones, but sex hormones (2) and cytokines such as tumor necrosis factor α (TNFα) and interleukins IL-1β and IL-6, have been identified as modulators of the HPA axis (3, 4). The functional connection between the immune system and HPA axis is essential for appropriate responses to homeostatic threats due to bacterial and viral infections (5). The activation of the HPA axis results in the release of corticosteroids from the adrenal cortex, principally cortisol in humans and corticosterone (CORT) in rats and mice (3). Moreover, other adrenal steroids, such as progesterone (PROG) and 11-deoxycorticosterone (DOC), are released (6–8) together with their neuroactive derivatives, which exert protective or adverse effects on neural tissues (9). These derivatives are synthesized from PROG and DOC by 5α-reduction into 5α-dihydroprogesterone and 5α-dihydrodeoxycorticosterone, which are further converted by 3α-hydroxysteroid dehydrogenase (3α-HSD) to 3α,5α-tetrahydroprogesterone (allopregnanolone, 3α,5α-THP) and 3α,5α-tetrahydrodeoxycorticosterone (3α,5α-THDOC) (9). The major source of these steroids seems to be the adrenal glands (10–12), even if some of them are also synthetized directly in the brain (9).

The immediate control of plasma concentrations of steroids derived from the adrenal gland and other tissues depends not only on *de novo* synthesis from cholesterol but also on the routes of the clearance of these steroids, which occur predominantly through A-ring reductases (5α/5β-reductase), 20α-hydroxysteroid dehydrogenases (20α-HSD) (13), and 11β-hydroxysteroid dehydrogenase type 2 (11β-HSD2) (14). In some tissues, 11β-HSD2 inactivates CORT, cortisol and other 11-hydroxysteroids to 11-dehydrocorticosterone (11-dehydroCORT), cortisone and other 11-ketosteroids, and this removal is offset by the opposite reaction catalyzed by 11β-hydroxysteroid dehydrogenase type 1 (11β-HSD1), which is expressed predominantly in the liver and adipose tissue (14). In humans, the regeneration of cortisol from cortisone within the splanchnic bed is equal to if not greater than that produced by nonsplanchnic tissues (e.g., the adrenals) (15), and at least in dogs, this process takes place primarily in the liver (16).

**Figure 1:**
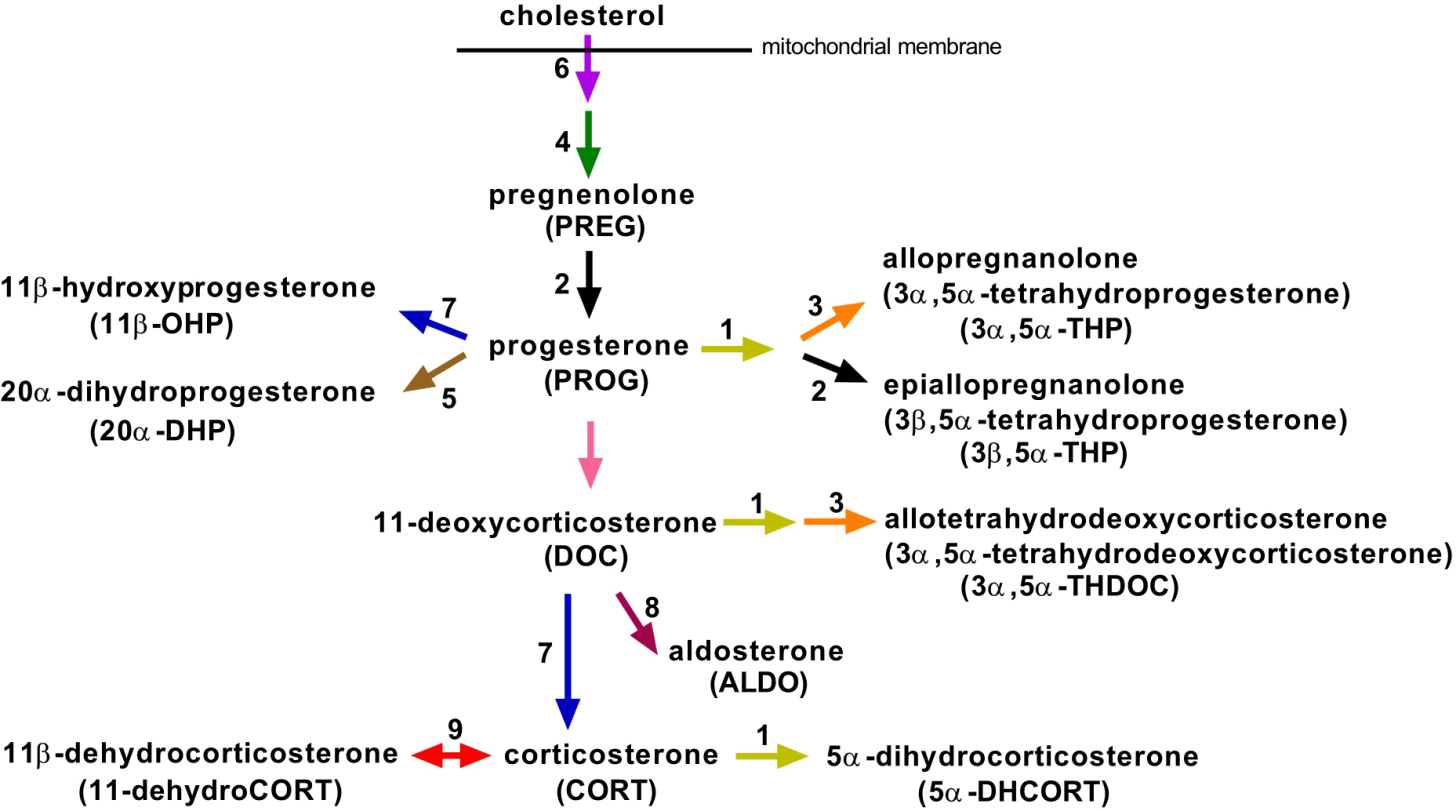
Overview of the main steroidogenic pathways involved in the biosynthesis of progesterone and corticosteroids and their metabolites, which we identified in mouse plasma. Enzymes and regulatory factors that may catalyze the respective reaction and that were studied in this work are marked with an Arabic numeral: 1, 5α-reductases (*Srd5a1*, *Srd5a3*); 2, 3β-hydroxysteroid dehydrogenases (*Hsd3b1*, *Hsd3b2*, *Hsd3b3*, *Hsd3b4*, *Hsd3b5*, *Hsd3b6*); 3, 3α-hydroxysteroid dehydrogenases (*Akr1c14*, *Dhrs9*, *Akr1c21*); 4, cholesterol side-chain cleavage enzyme (*Cyp11a1*); 5, 20α-hydroxysteroid dehydrogenases (*Akr1c18*, *Akr1c6*, *Hsd17b1*, *Hsd17b2*); 6, steroidogenic acute regulatory protein (*Star*); 7, 11β-hydroxylase (*Cyp11b1*); 8, aldosterone synthase (*Cyp11b2*); 9, 11β-hydroxysteroid dehydrogenases (*Hsd11b1*, *Hsd11b2*).

Ample evidence shows that HPA activity in response to stressful situations is modulated by inflammatory-related factors (4) and by gut microbiota (17–19), although some inconsistencies, possibly due to different stress stimuli, have been reported. The interaction between microbiota and brain is bidirectional using several different mechanisms of communication (20). Chronic stress results in pronounced alterations of the gut microbial community (21, 22), whereas acute stress results in transcriptional reprogramming of the bacteria with minimal changes in their relative abundance (23). Additionally, the gut microbiota impacts the metabolism of endogenous steroids (24) and the concentration of neuroactive steroids in the brain (25). Because these studies indicate a link between commensal bacteria, activity of the HPA axis and steroid metabolism, investigating whether activation of the HPA axis and corticosteroid metabolism depend on microbial colonization is worthwhile. Therefore, the aim of the following study was to assess the effect of acute physical (immune) stressor on the steroid response (**Figure 1**) in germ-free (GF) and specific pathogen-free (SPF) mice. Acute immune stress was realized by a single administration of anti-CD3 antibody, which leads to a rapid temporary upregulation of cytokines associated with the activation of T cells and macrophages (26) and with the modulation of steroidogenesis (27–30).

## Materials and methods

### Animals, treatment, and tissue collection

The experiments were performed on 9 to 13 weeks-old GF and SPF male BALB/c mice (Institute of Microbiology, Nový Hrádek, Czech Republic) kept on a 12-hour light/dark cycle with unrestricted access autoclaved tap water and 50 kGy irradiated sterile pellet (diet no. 1414, Altromin, Lage, Germany) ad libitum. GF mice were derived from the SPF BALB/c mice by Cesarean section and kept under axenic conditions in Trexler-type plastic isolators for at least 20 generations. The sterility was controlled every 2 weeks by confirming the absence of bacteria, molds, and yeast by aerobic and anaerobic cultivation of mouse feces and swabs from the isolators in VL (Viande-Levure), Sabouraud-dextrose and meat-peptone broth and subsequent plating, and aerobic/anaerobic cultivation on blood, Sabouraud and VL agar plates. The GF status of the mice was further confirmed by the cecal size and weight when GF mice were used in the experiments (31). Pathogen-free status of SPF mice was monitored by standard microbiological control according to FELASA. To ensure equal conditions for GF and SPF mice, SPF mice received the same autoclaved drinking water and pelleted diet, were reared on sterile bedding and were handled by the same staff as the GF mice. One day before the experiment, all mice were transferred into sterile individually ventilated cages equipped with a filter system (IVC box, Tecniplast S.p.A., Buguggiate, Italy) to minimize contamination of GF mice with microorganisms and to ensure the same conditions for both GF and SPF mice.

Immune stress was induced according to the method of Cima et al. (32) by injection of the anti-CD3 antibody, which activates the adaptive immune response associated with a systemic rise in corticosterone. Both GF and SPF mice received an i.p. injection of 100 μl of saline containing 10 μg of the anti-CD3 antibody (145-2C11, eBioscience, purchased from Thermo Fisher Scientific, cat. no. 16-0031-85) in a laminar flow hood and were left in sterile IVC boxes. Control animals received saline without anti-CD3. Tissues were collected 4 hours after the injection. Mice were rapidly anesthetized with isoflurane vapor, blood was collected by cardiac puncture and centrifuged, and the plasma was stored at -80°C. Then, the mice were euthanized by decapitation, and the adrenal glands, liver, colon and testes were harvested, immediately frozen on dry ice, stored at -80°C and kept in these conditions until processing. To minimize the effect of diurnal factors, the mice were injected with the anti-CD3 antibody and saline between 9:00 and 11:00 a.m. The experiments were approved by the Animal Care Committee of the Institute of Microbiology, Czech Academy of Sciences.

### Steroid extraction from plasma

Steroids were extracted from mice plasma using an optimized protein precipitation protocol described by Gazárková et al. (33). Here, 40 µl of plasma were spiked with 2 µl of internal standards solution (50 µg/ml) and precipitated with 200 µl of acetonitrile, vortexed for 3 s, and incubated for 10 minutes at laboratory temperature. Next, the samples were centrifuged at 17,530 x g and 4°C for 10 minutes (Hettich Mikro 220R, Hettich, Germany). The supernatant was transferred to a clean Eppendorf tube and evaporated under vacuum at 60°C for 40 minutes (Vacuum Concentrator Plus, Eppendorf™, Germany). The dried residue was reconstituted prior to UHPLC-MS/MS analysis in 40 µl of 50% acetonitrile, mixed at 1400 rpm for 15 minutes at laboratory temperature (Thermo-Shaker TS-100 C, Biosan, Latvia), and transferred into the vial for LC-MS/MS analysis.

### Analysis of plasma steroids by UHPLC-MS/MS

The UHPLC-MS/MS analysis developed for endogenous steroid profiling followed the protocol described by the study of Gazárková et al. (33). Briefly, the chromatographic separation was carried out on an Acquity UPLC I-Class system (Waters Corp., Milford, MA, USA) using a reversed-phase CortecsC18 column (2.1 x 150 mm, 1.6 µm) protected with Acquity Column In-Line Filter, both Waters Corp. (Milford, MA, USA). Steroids were chromatographically resolved by gradient elution of 0.1% formic acid in water and acetonitrile in 20 minutes. The Xevo TQ-XS spectrometer (Waters Corp., Milford, MA, USA) was operated with an electrospray ionization source (ESI) in positive and negative ion modes with the following source conditions: capillary voltage 2.3 kV (ESI+) and 0.5 kV (ESI-), ion source temperature 150°C, desolvation temperature 650°C, cone gas (nitrogen) flow 240 l/h, desolvation gas (nitrogen) flow 900 l/h, nebulizer gas (nitrogen) pressure 7.0 bar. Data were acquired using MassLynx V4.2 and processed using TargetLynx XS software. Quality control samples were measured once per 12 hours to ensure proper performance of UHPLC-MS/MS analyses. Steroid concentrations were extrapolated from calibration curves and converted to ng/ml. The method showed good linearity (R^2^>0.99) and the average recovery between 80-90% for all analytes. Limits of quantification (LOQ) ranged from 0.1 to 3.0 ng/ml, except for corticosterone with LOQ 30 ng/ml, as a result of strict EMA validation criteria for matrix effects, accuracy, and precision. Detailed parameters of the method validation are reported in our submitted study (33).

Using above described method we analyzed these steroids: pregnenolone (PREG), progesterone (PROG), 11-deoxycorticosterone (DOC), corticosterone (CORT), aldosterone (ALDO), allopregnanolone (3α,5α-tetrahydroprogesterone, 3α,5α-THP), epiallopregnanolone (3β,5α-tetrahydroprogesterone, 3β,5α-THP), allotetrahydro-deoxycorticosterone (3α,5α-THDOC), 20α-dihydroprogesterone (20α-DHP), 11β-hydroxyprogesterone (11β-OHP), 5α-dihydrocorticosterone (5α-DHCORT), 11-dehydrocorticosterone (11-dehydroCORT).

If the value was under the LOQ, one-third of the LOQ value was used for the statistical calculations and graphic visualization. The values under detection limit were set to zero.

### RNA extraction, cDNA synthesis and real-time polymerase chain reaction

Total RNA was isolated from whole frozen adrenal glands or 30-50 mg of frozen liver, colon, and testis tissue using an RNeasy Plus Universal Mini Kit (Qiagen, Hilden, Germany) according to the manufacturer’s instructions. The concentration and purity of RNA was determined by a NanoDrop spectrophotometer (NanoDrop Technologies, Wilmington, DE, USA). cDNA was synthesized using random hexamers and the High Capacity cDNA Reverse Transcription Kit (Life Technologies, Carlsbad, Ca, USA), and the cDNA samples were analyzed by real-time polymerase chain reaction using a LightCycler 480 PCR System (Roche Diagnostic GmbH, Mannheim, Germany). Relative expression levels of the genes of interest and reference genes were assessed using 5x Hot Firepol Probe QPCR Mix Plus (ROX) (Solis BioDyne, Tartu, Estonia) and TaqMan probes specific for the studied transcripts (TaqMan Assays, Life Technologies). The following TaqMan probes were used: steroidogenic acute regulatory protein StAR/STARD1 (*Star*, Mm00441558_m1); cholesterol side-chain cleavage enzyme, P450scc (*Cyp11a1*, Mm00490735_m1); 3β-hydroxysteroid dehydrogenases type 1 to 6 (*Hsd3b1*, Mm01261921_mH; *Hsd3b2*, Mm00462685_m1, *Hsd3b3*, Mm01729523_m1; *Hsd3b4*, Mm00843753_s1; *Hsd3b5*, Mm00657677_mH; *Hsd3b6*, Mm00834440_m1); 11β-hydroxylase (*Cyp11b1*, Mm01204952_m1); aldosterone synthase (*Cyp11b2*, Mm00515624_m1); 3α-hydroxysteroid dehydrogenases (*Akr1c14*, Mm00506338_m1; *Dhrs9*, Mm00615706_m1); 20α-hydroxysteroid dehydrogenase (*Akr1c18*, Mm00506289_m1); 17α-hydroxysteroid dehydrogenases, which possess also 3α- (*Akr1c21*, Mm00472624_m1) or 20α-activity (*Hsd17b1*, Mm00501692_g1; *Hsd17b2*, Mm00500430_m1; *Hsd17b5* (*Akr1c6*, Mm00662937_m1); 5α-reductase type 1 (*Srd5a1*, Mm00614213_m1); 5α-reductase type 3 (*Srd5a3*, Mm00491099_m1); 11β-hydroxysteroid dehydrogenase type 1 (*Hsd11b1*, Mm00476182_m1) and 11β-hydroxysteroid dehydrogenase type 2 (*Hsd11b2*, Mm01251104_m1). In addition, the expression of liver receptor homolog 1 (*Nr5a2*, Mm00446088_m1), a regulatory factor of intestinal extra-adrenal steroidogenesis (34), cochaperone FK506 binding protein 5 (*Fkbp5*, Mm00487401_m1), a glucocorticoid-sensitive marker (35), and the proinflammatory cytokines tumor necrosis factor α (*Tnfα*, Mm00443258_m1), interleukin-1β (*IL1β*, Mm00434228) and interleukin-6 (*IL6*, Mm00446190) were examined. The expression levels of the genes of interest in the adrenal gland were normalized to those of peptidylprolyl isomerase B (*Ppib*, Mm00478295_m1) and succinate dehydrogenase subunit A (*Sdha*, Mm01352366_m1), those in the colon and testis to *Ppib* and hypoxanthine-guanine phosphoribosyltransferase 1 (*Hprt*, Mm01545399_m1) and those in the liver to *Hprt1* and phosphoglycerate kinase 1 (*Pgk1*; Mm00435617_m1). The selected reference genes provided the highest stability in the panel of 6 potential reference genes tested. The quantity of the PCR products was determined using the standard calibration curve method with 3-fold dilutions of the mixed cDNA from samples. The genes were considered not detectable if there was no signal at all or if the crossing point (Cp) of 3-fold diluted cDNA was higher than 36.

### Western blot analysis

Samples of adrenal tissue were homogenized in 90 μl T-Per buffer (Thermo Fisher Scientific) containing protease inhibitor cocktail (1x; Roche Diagnostics GmbH, Mannheim, Germany) using an Ultrasonic processor UP100H (Hielscher Ultrasonics GmbH, Germany). Homogenates were centrifuged at 18,000 x g for 10 min at 4°C, and supernatants were collected and stored at -80°C in two aliquots. Protein concentration was measured in the first aliquots by the Coomassie blue method, whereas the second aliquot was diluted to 0.5 µg/µl using T-Per buffer containing protease inhibitor cocktail and 4x Laemmli sample buffer (Bio-Rad Laboratories, Inc., CA, USA) containing 10% 2-mercaptoethanol, heated at 70°C for 10 min under constant shaking, and stored at -20°C.

Proteins (5 μg) were separated by 10% SDS-PAGE gels (TGX Stain-free FastCast Acrylamide kit) and TGS buffer (1x) using the Mini-PROTEAN Tetra Cell system (200 V; Bio-Rad Laboratories). After stain-free activation (ChemiDoc Touch Imaging System, Bio-Rad) the proteins were electrophoretically transferred to a methanol-activated PVDF membrane (Immobilon-FL, 0.45 μm, Merck Millipore, Darmstadt, Germany) using TG buffer (1x; with addition of 20% methanol and 0.037% SDS) and semidry blotting system according to a standard protocol (25 V, 1 A, 30 min; Trans-Blot Turbo Transfer System, Bio-Rad). Efficient transfer was verified in gels and membranes by a stain-free method. The membranes were blocked in blocking buffer (10% SEA Block, Thermo Fisher Scientific) diluted in TBST buffer for 30 min at room temperature (RT) and then incubated with IRDye 800CW donkey anti-rabbit IgG secondary antibody (LI-COR, Lincoln, NE, USA; 1:20,000 in 10% SEA buffer, RT, 60 min). The membranes were washed five times for 5 min in TBST buffer and twice for 5 min in TBS buffer, and captured on the ChemiDoc system to detect cross reactions of secondary antibody. Subsequently, the membranes were cut into strips, and parts with molecular weights of proteins between 20 and 37 kDa were incubated with anti-StAR antibody (ab96637, Abcam, Cambridge, UK; 1:4,000 in 10% SEA buffer, 4°C, overnight) and parts between 37 and 150 kDa with CYP11a1 antibody (ASA-B0541, Novatein Biosciences, MA, USA; 0.125 μg/ml in 10% SEA buffer, 4°C, overnight). The following day, the membranes were washed five times in TBST buffer and incubated again with IRDye 800CW donkey anti-rabbit IgG secondary antibody at RT for 60 min, washed five times in TBST buffer and twice in TBS buffer and captured on a ChemiDoc system to detect the signal for StAR and CYP11a proteins.

The membranes with protein between 37-150 kDa were also used to determine 3β-HSD1,2 and β-actin as an internal standard. First, the membranes were washed twice in TBST buffer, incubated with IRDye 680CW donkey anti-mouse IgG secondary antibody (LI-COR, Lincoln, NE, USA; 1:20,000 in 10% SEA buffer, RT, 60 min), washed five times in TBST buffer and twice in TBS buffer, and captured on a ChemiDoc system to detect cross reactions of the secondary antibody. Subsequently, the membranes were incubated with 3β-HSD1,2 antibody (sc-51520, Santa Cruz Biotechnology, Inc., TX, USA; 1:5,000 in 10% SEA buffer, 4°C, overnight). The following day, the membranes were washed five times in TBST buffer and incubated again with IRDye 680CW donkey anti-mouse IgG secondary antibody at RT for 60 min, washed five times in TBST buffer and twice in TBS buffer, and captured on a ChemiDoc system to detect the signal for 3β-HSD1,2 protein. Then, the membranes were washed twice in TBST buffer and reprobed with monoclonal β-actin antibody (A1978, Sigma-Aldrich, Darmstadt, Germany; 1:10,000 in 10% SEA buffer, RT, 60 min), washed five times in TBST buffer and incubated again with IRDye 680CW donkey anti-mouse IgG secondary antibody at RT. After 30 min the membranes were washed five times in TBST buffer and twice in TBS buffer and captured on a ChemiDoc system to detect the signal for β-actin protein. The results were calculated as a ratio of fluorescent signals of studied proteins and β-actin. We also tried to measure the level of 11β-hydroxylase using a CYP11B1 polyclonal antibody (BS-3898R, Bioss Antibodies, MA, USA), but unfortunately, we were unable to detect any signal for this protein.

### Data analysis

Statistical analysis was performed using GraphPad Prism 6 software (GraphPad, La Jolla, CA, USA). Data were assessed for normality using the Shapiro-Wilk test and for equality of variances using the Brown-Forsythe test. When necessary, the data were transformed to fit assumptions of normality and homogeneity of variance prior to analysis. Data in graphs are nontransformed. Comparison of more than two groups was performed by two-way ANOVA with microbial status and treatment as the two main factors followed by Tukey’s multiple comparisons test for Gaussian distribution, whereas Kruskal-Wallis test followed by Dunn’s multiple comparison test was used for non-Gaussian distribution. For comparing two groups, Student’s t-test was used. All data are expressed as the means ± SEM.

## Results

### Acute immune stress modulates plasma levels of steroids

**Figure 2:**
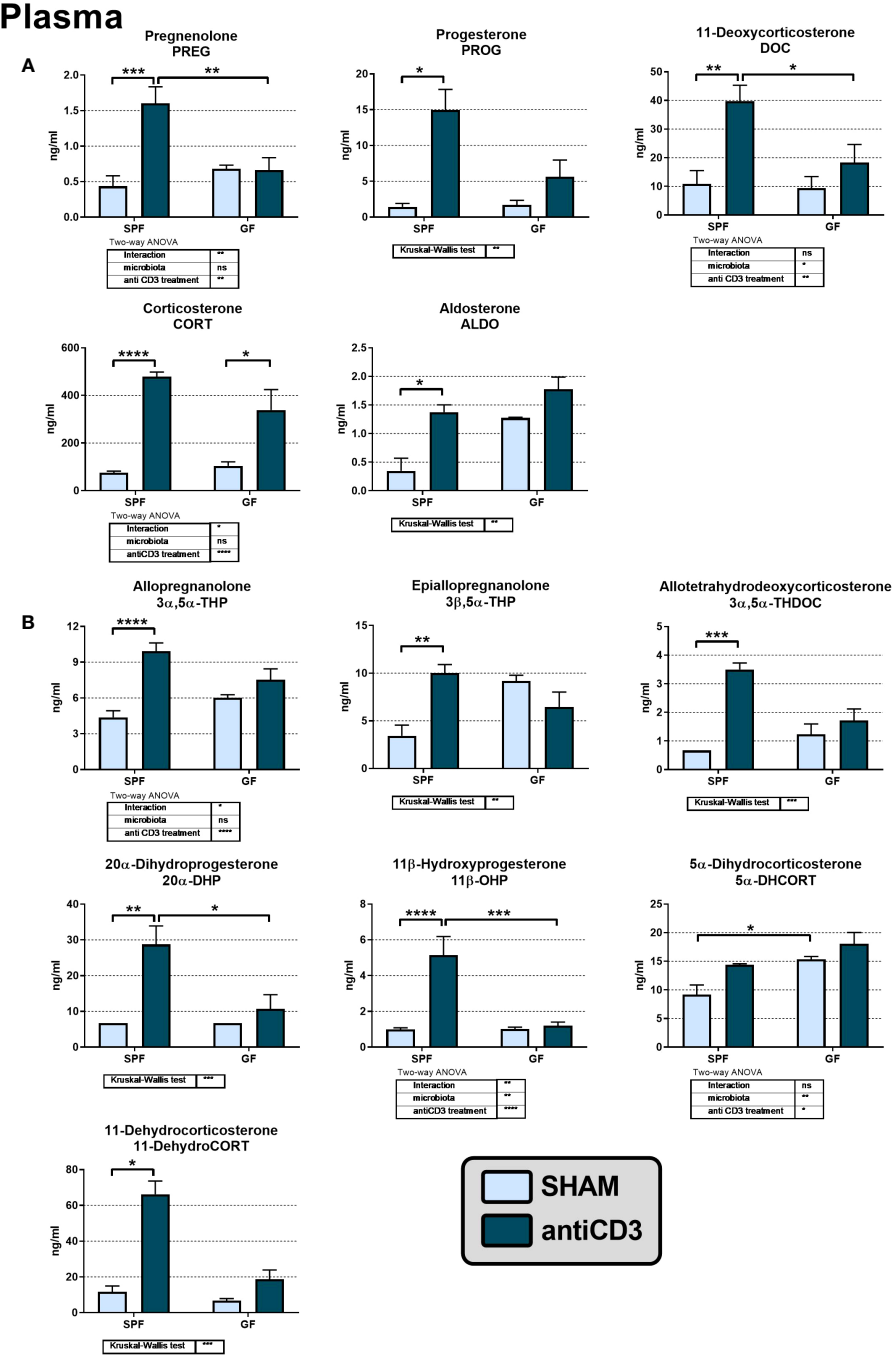
The levels of adrenal steroids **(A)** and their metabolites **(B)** in the plasma of control and anti-CD3 treated SPF and GF mice. Blood was collected 4 hours after i.p. injection of 100 μl of saline containing 10 μg of the anti-CD3 antibody (antiCD3) or saline alone (SHAM). The results are presented as the means ± SEM (n = 4 - 9 per group) and were analyzed by a two-way ANOVA followed by Tukey’s *post hoc* test or by Kruskal-Wallis ANOVA followed by Dunn’s *post hoc* test. The results of two-way ANOVA and Kruskal-Wallis ANOVA are given in tables, and the results of *post hoc* tests are given in the histograms: ^*^P < 0.05; ^**^P < 0.01; ^***^P < 0.001; ^****^P < 0.0001. The missing SEM above a column means, that all values of the group were below the limit of quantification (LOQ; see materials and methods). If the value was under the LOQ, one-third of the LOQ value was used for the statistical calculations and graphic visualization. ns, not significant.

To investigate the effect of microbiota on the activation of the HPA axis and steroid response to stress, we measured the profile of plasma corticosteroids and their precursors and metabolites in SPF and GF mice exposed to immune stress by anti-CD3 antibody injection. As shown in **Figure 2**, microbiota sensitized the steroid response to acute immune stress. Stress challenge increased the plasma levels of steroids of the corticosteroid synthetic pathway regardless of steroid type but only in SPF mice (**Figure 2A**). In GF mice, anti-CD3 had no significant effect on the plasma levels of these steroids except corticosterone (**Figure 2A**).

Microbiota also affected the levels of the pregnane metabolites of PROG and DOC, which belong to the group of neuroactive steroids. Anti-CD3 significantly elevated 3α,5α-THP, 3β,5α-THP and 3α,5α-THDOC, but only in SPF and not in GF mice (**Figure 2B**). Basal levels of neuroactive steroids in unstressed GF mice did not differ significantly from their SPF counterparts even if the level of 3β,5α-THP was 2.6-fold higher in GF than in SPF mice (P = 0.055). Regarding other PROG and CORT metabolites, we identified 20α-DHP, 11β-OHP, 5α-DHCORT and 11-dehydroCORT in plasma and the levels of these metabolites were, except for 5α-DHCORT, significantly increased after stress challenge but only in the presence of microbiota (**Figure 2B**).

### Increased corticosteroid levels in SPF mice treated with anti-CD3 are not associated with increased expression of mRNA and proteins of adrenal steroidogenesis

**Figure 3:**
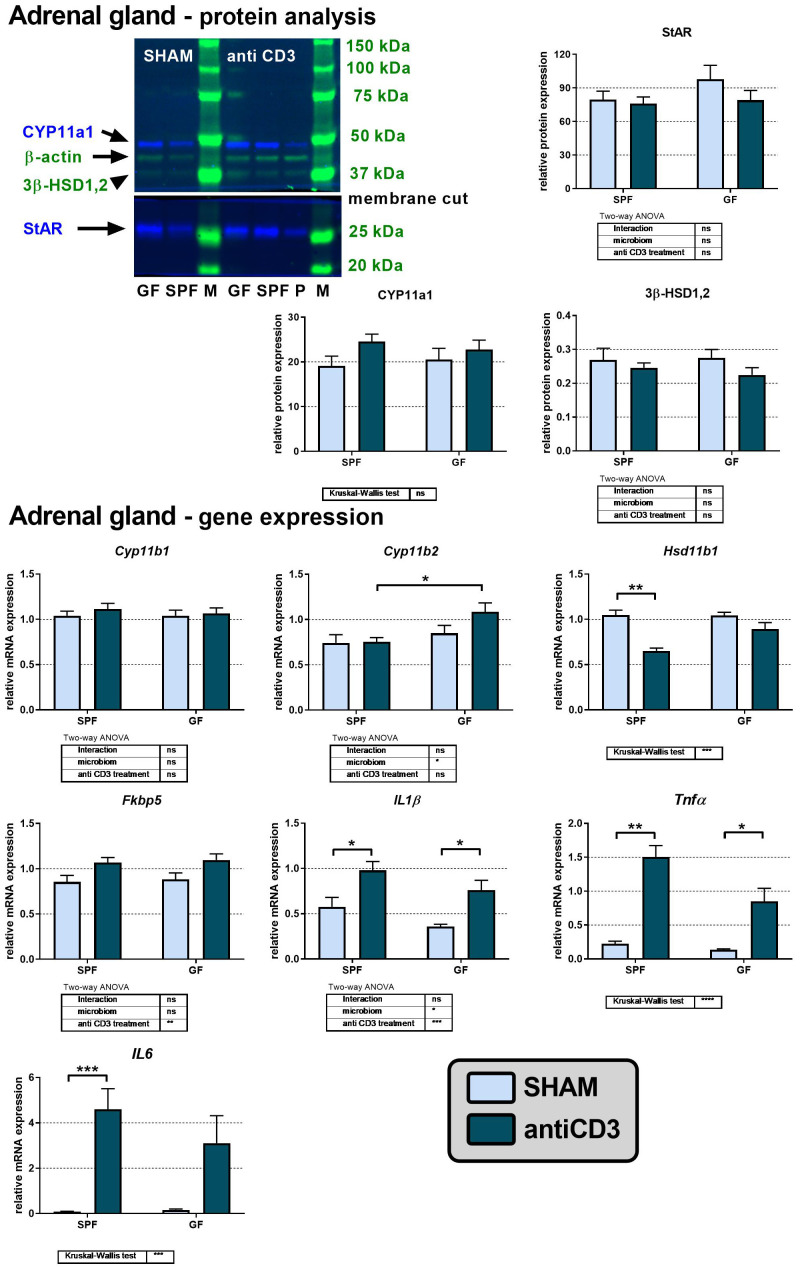
Effect of anti-CD3 treatment on protein and mRNA levels of steroidogenic enzymes and acute regulatory protein in adrenal glands of SPF and GF mice. The western blot image shows the detection and analysis of the targeted proteins. The relative expression of protein and mRNA was measured 4 hours after i.p. injection of 100 μl of saline containing 10 μg of the anti-CD3 antibody (antiCD3) or saline alone (SHAM). The results are presented as the means ± SEM (n = 7 - 10 per group) and were analyzed by two-way ANOVA followed by Tukey’s *post hoc* test or by Kruskal-Wallis ANOVA followed by Dunn’s *post hoc* test. The results of two-way ANOVA and Kruskal-Wallis ANOVA are given in tables, and the results of *post hoc* tests in the histograms: *P < 0.05; **P < 0.01; ***P < 0.001, ****P < 0.0001; ns, not significant; P, positive control; M, protein marker.

The observation that plasma levels of corticosteroids and their precursors are elevated after anti-CD3 injection predominantly in SPF but not GF mice led us to investigate the effect of anti-CD3 treatment on the adrenal steroidogenic pathway (**Figure 3**). We therefore evaluated the protein expression of steroidogenic acute regulatory protein (StAR) and the steroidogenic enzymes cholesterol side-chain cleavage enzyme (CYP11a1) and 3β-hydroxysteroid dehydrogenase (3β-HSD1,2). The purchased antibodies against steroid 11β-hydroxylase 1 were nonfunctional (no band was observed after serial dilutions in different buffers); therefore, we quantified the expression of this enzyme and the following aldosterone synthase at the mRNA level instead of protein level. As reported in **Figure 3**, the protein expression of StAR, CYP11a1 and 3β-HSD1,2 was similar in SPF and GF mice and was not changed after anti-CD3 stimulus. Similarly, mRNA expression of *Cyp11b1* encoding 11β-hydroxylase, which catalyzes the final step of corticosterone synthesis, was independent of microbiota and immune stress, whereas in the case of *Cyp11b2* encoding aldosterone synthase, a weak effect of microbiota was observed. Moreover, in SPF mice, anti-CD3 significantly downregulated the expression of *Hsd11b1*, which encodes the steroidogenic enzyme 11β-HSD1; *Hsd11b2* was not detectable in the adrenal gland.

Because proinflammatory cytokines have been previously identified as modulators of *de novo* steroidogenesis or regeneration of biologically active steroids (4, 36), we measured the expression of genes encoding the adrenal mRNA expression of the proinflammatory cytokines *IL1β*, *Tnfα*, and *IL6.* Overall, we found a strong effect of anti-CD3 treatment on the transcripts of *IL1β*, *Tnfα*, and *IL6* in both SPF and GF mice, although in GF mice, the upregulation of *IL6* did not reach significance (**Figure 3**). A weak stimulatory effect of anti-CD3 was also observed on the expression of *Fkbp5*, a known glucocorticoid target gene, which encodes the cochaperone participating in the regulation of the glucocorticoid receptor complex upon binding of stress hormones (35).

### Steroid metabolism in the colon of anti-CD3 treated mice

**Figure 4:**
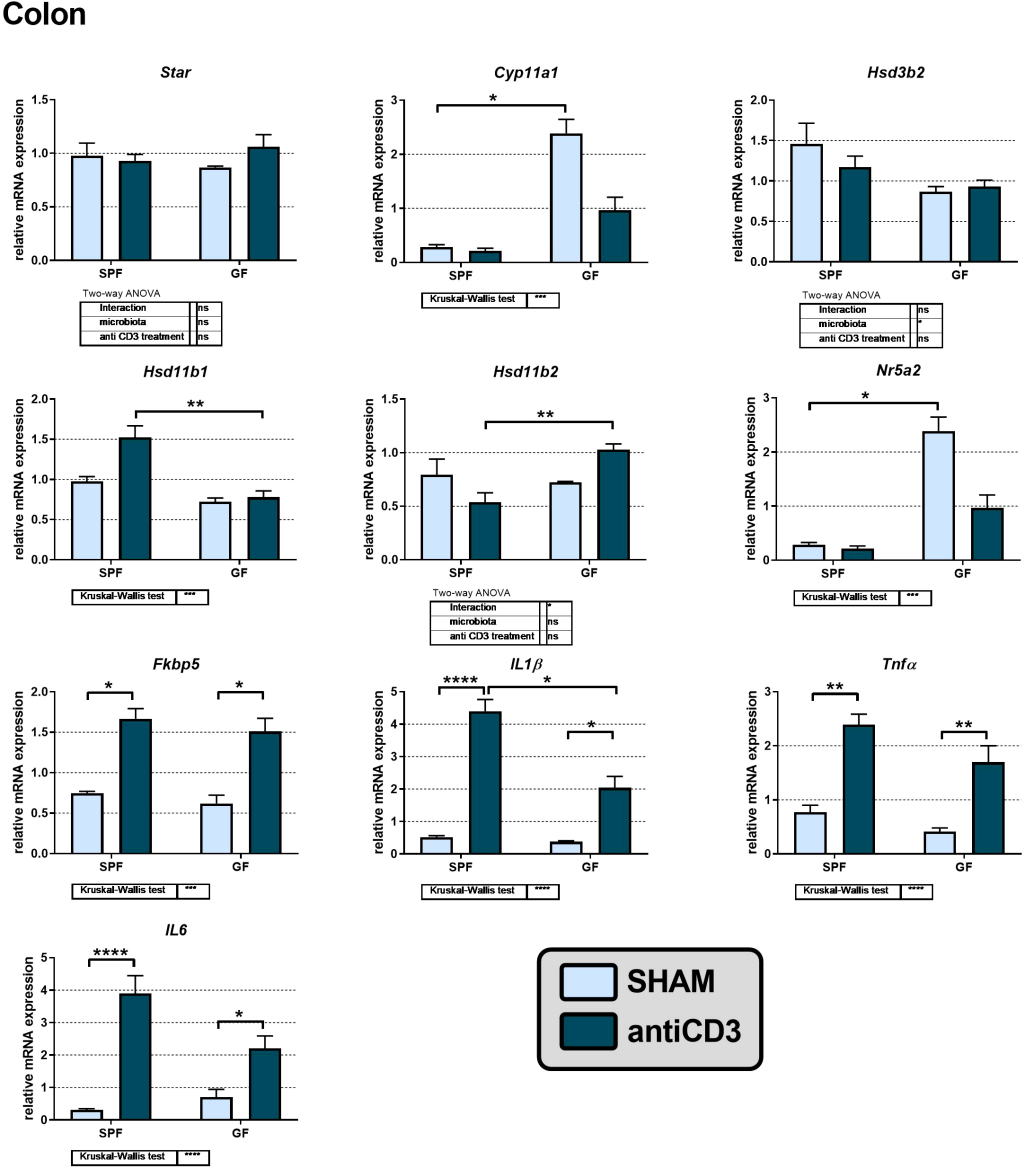
Effect of anti-CD3 treatment on the expression of genes of the steroidogenic pathway, proinflammatory cytokines and the glucocorticoid target gene *Fkbp5* in the colon of SPF and GF mice. The relative expression of mRNA was measured 4 hours after i.p. injection of 100 μl of saline containing 10 μg of the anti-CD3 antibody (antiCD3) or saline alone (SHAM). The results are presented as the means ± SEM (n = 4 - 18 per group) and were analyzed by two-way ANOVA followed by Tukey’s *post hoc* test or by Kruskal-Wallis ANOVA followed by Dunn’s *post hoc* test. The results of two-way ANOVA and Kruskal-Wallis ANOVA are given in tables, and the results of *post hoc* tests are given in the histograms: *P < 0.05; **P < 0.01; ***P < 0.001, ****P < 0.0001; ns, not significant.

In peripheral tissues, including the murine colon, glucocorticoid synthesis was considered to be limited to corticosterone reactivation by 11β-HSD1 following its prior inactivation by 11β-HSD2. However, some findings demonstrated the capability of extra-adrenal *de novo* steroidogenesis in the small and large intestine (32, 37) and the effect of microbiota on local synthesis of CORT in the small intestine (38). To identify whether acute immune stress modulates steroidogenic pathways in the adrenal gland and extra-adrenal organs differently, we investigated the colon response to anti-CD3 in the presence or absence of gut microbiota. We identified the expression of genes encoding the early steps of steroidogenesis, specifically *Star*, *Cyp11a1* and *Hsd3b2*, but not the expression of *Cyp11b1*, which is responsible for the conversion of DOC to CORT. In contrast, *Hsd11b1* encoding the enzyme 11β-HSD1, which regenerates CORT from biologically inactive 11-dehydroCORT, was expressed in the colon together with *Hsd11b2* encoding the enzyme 11β-HSD2, which operates in the opposite direction. As shown in **Figure 4**, microbiota downregulated the expression of *Cyp11a1*, encoding the rate-limiting steroidogenic enzyme P450scc, and *Nr5a2*, encoding a regulatory factor of intestinal extra-adrenal steroidogenesis (34). Acute immune stress also upregulated the expression of *Hsd11b2* in GF compared to that in SPF mice, whereas *Hsd11b1* was upregulated in SPF compared to that in GF animals, suggesting increased local regeneration and decreased inactivation of CORT in the colon of stressed SPF mice.

To determine whether the colon was activated in response to anti-CD3 treatment, we measured the adrenal mRNA expression of the proinflammatory cytokines *IL1β*, *Tnfα*, and *IL6* and *Fkbp5*. As shown in **Figure 4**, anti-CD3 treatment upregulated the expression of all three cytokines, and in the case of *IL1β* the effect of anti-CD3 was significantly higher in SPF mice than in GF mice. The glucocorticoid target gene *Fkbp5* was also upregulated after anti-CD3 treatment. Overall, this effect of anti-CD3 treatment on the expression of colonic proinflammatory cytokines and *Fkbp5* was very similar to what we observed in the adrenal glands. Similarly, we did not find upregulation of the first steps of steroidogenesis (*Star*, *Cyp11a1*, *Hsd3b*) either in the adrenal gland or in the colon after immune stress, but in the colon, we observed significant downregulation of genes encoding the rate-limiting step of steroidogenesis (*Cyp11a1*) and the regulatory factor of intestinal steroidogenesis (*Nr5a2*) by microbiota.

### Acute immune stress disturbs the transcription profile of steroid machinery elements in the testis

The profiling of plasma steroids (**Figure 2**) showed that acute immune stress upregulated plasma steroids predominantly in SPF animals but without a clear effect on enzymes of adrenal steroidogenesis. To explore whether this finding might reflect different broader steroid machinery response to stress, we further analyzed the expression of genes encoding enzymes that catalyze *de novo* synthesis of C_21_-steroids in the testis and the metabolism of 5α-, 3α/β- and 20α-reduced steroids in the testis and liver of SPF mice because the testis is one of the sources of plasma PROG in animals exposed to acute stress (7) and PROG, DOC and CORT are good substrates for 5α-reductases and 3α/3β- and 20α-HSDs expressed in these organs (13). Analysis of steroid enzymes expression was restricted only to SPF mice because anti-CD3 stimulus modulated steroid plasma levels preferentially in SPF and not in GF mice.

**Figure 5:**
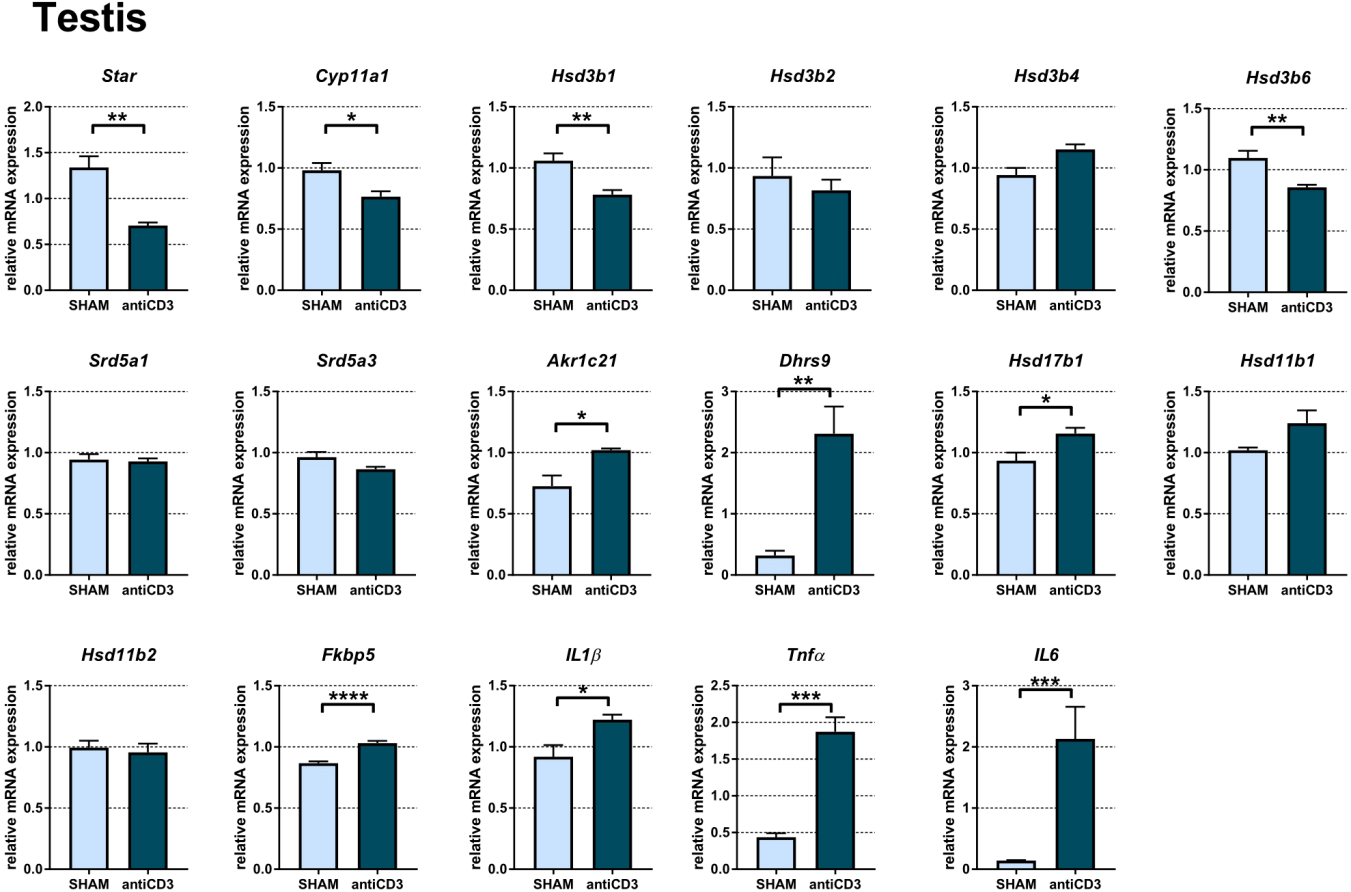
Expression of genes of steroidogenic pathways, proinflammatory cytokines and the glucocorticoid target gene *Fkbp5* in the testes of SPF mice treated with anti-CD3. The relative expression of mRNA was measured 4 hours after i.p. injection of 100 μl of saline containing 10 μg of the anti-CD3 antibody (antiCD3) or saline alone (SHAM). The results are presented as the means ± SEM (n = 7 - 8 per group) and were analyzed by unpaired Student’s *t*-test: *P < 0.05; **P < 0.01; ***P < 0.001, ****P < 0.0001.

Analysis of the testis revealed that acute immune stress resulted in downregulation of genes involved in gonadal steroidogenesis. As shown in **Figure 5**, *Star*, *Cyp11a1*, *Hsd3b1*, and *Hsd3b6* encoding the first steps of PROG synthesis, were downregulated, which indicates decreased PROG synthesis in testes exposed to immune stress. For other murine isoforms of 3β-HSDs, which function predominantly as dehydrogenase/isomerases (*Hsd3b2*, *Hsd3b3*) or exclusively as 3-ketosteroid reductases (*Hsd3b4*, *HSD3b5*), the transcripts of *Hsd3b3* and *Hsd3b5* were not detected, and the expression of *Hsd3b2* and *Hsd3b4* was not modulated by anti-CD3 treatment. The expression of *Hsd3b2* was very low (Cp > 35), much lower than that of *Hsd3b4*, *Hsd3b1 and Hsd3b6*. As steroids with reduced Δ_4,5_ bond and 3α- and 20α-oxo-groups were increased in the plasma of stressed SPF mice, we further tested whether immune stress modulates the expression of genes encoding enzymes with 5α-reductase and 3α- and 20α-HSD activities (**Figure 5**). The expression of *Srd5a1* and *Srd5a3*, the genes encoding 5α-reductases, was not altered following acute stress. In contrast, the transcription profile of genes (*Akr1c14*, *Akr1c21*, *Dhrs9*) encoding enzymes with 3α-HSD activity was different. Whereas *Akr1c21* and especially *Dhrs9* were upregulated in anti-CD3 treated mice, *Akr1c14* was expressed in the testis but could not be quantified due to the impossibility constructing a three-point calibration curve with PCR efficiency of at least 1.8-2.2. Of the genes encoding enzymes with 20α-HSD activities (*Akr1c6*, *Akr1c18*, *Hsd17b1*, *Hsd17b2*), we found only the transcript of *Hsd17b1*, which was slightly increased after anti-CD3 application. All other transcripts were considered not detectable. To identify the effect of immune stress on the immediate control of glucocorticoids, we quantified the transcripts of key enzymes of three pathways responsible for the control of glucocorticoids – i) *de novo* synthesis (*Cyp11b1*, not detectable), ii) regeneration of glucocorticoids from inactive metabolites (*Hsd11b1*), and iii) inactivation of glucocorticoids (*Hsd11b2*). As shown in **Figure 5**, the expression of *Hsd11b1* and *Hsd11b2* remained unchanged during acute immune stress even if stress concomitantly induced markers of glucocorticoid activation (*Fkbp5*) and inflammation (*IL1β*, *Tnfα*, *IL6*).

### Acute immune stress modulates steroidogenic pathways associated with the synthesis of reduced steroids in the liver, adrenal gland and colon

**Figure 6:**
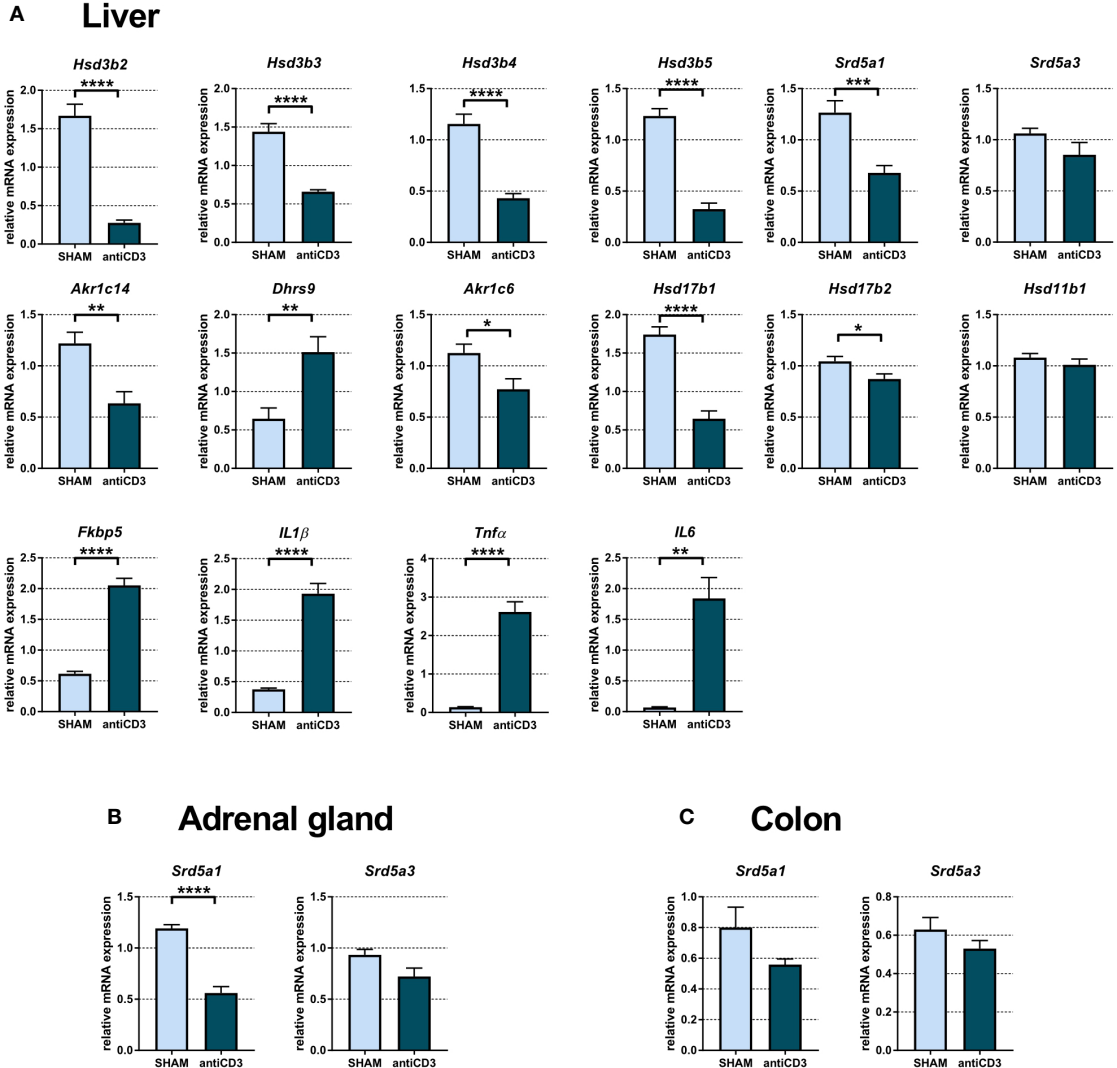
Expression of genes of steroidogenic pathways, proinflammatory cytokines and the glucocorticoid target gene *Fkbp5* in liver **(A)**, adrenal gland **(B)** and colon **(C)** of SPF mice treated with anti-CD3. The relative expression of mRNA was measured 4 hours after i.p. injection of 100 μl of saline containing 10 μg of the anti-CD3 antibody (antiCD3) or saline alone (SHAM). The results are presented as the means ± SEM (n = 7 - 9 per group) and were analyzed by unpaired Student’s *t*-test: *P < 0.05; **P < 0.01; ***P < 0.001, ****P < 0.0001.

It is well known that the liver is an organ that does not have the ability to transform cholesterol into active steroids, but depending on the expressed enzymes, the liver produces steroid metabolites from various steroid precursors. Therefore, we analyzed the mRNA expression of steroid 5a-reductases and dehydrogenases with 3α/β-, 11β-, and 20α-activities. Analysis of hepatic mRNAs indicated that anti-CD3 injection led to a significant downregulation of transcripts of genes encoding enzymes possessing 3α- (*Akr1c14*), 3β- (*Hsd3b2*, *Hsd3b3*, *Hsd3b4*, *Hsd3b5*), 5α- (*Srd5a1*) and 20α-HSD (*Akr1c6*, *Hsd17b1*, *Hsd17b2*) activities, with the exception of *Srd5a3* and *Hsd11b1*, which were not changed, and *Dhrs9*, which was upregulated (**Figure 6A**). *Dhrs9*, which encodes oxidative 3α-HSD, in contrast to *Akr1c14*, was upregulated in the liver of anti-CD3 treated SPF, similar to *IL1β*, *Tnfα*, *IL6*, and *Fkbp5*. Transcripts of *Hsd3b6*, *Akr1c18*, *Akr1c21* and *Hsd11b2* were not detectable and the level of *Hsd3b1* was very low (Cp > 36).

We next examined the expression of genes encoding steroid enzymes involved in the 5α-reduction of PROG, DOC and CORT in the adrenal gland and colon. Since *Cyp17a1*, encoding 17α-hydroxylase/17,20 lyase, is absent in mouse adrenals, PROG, which is not used for the synthesis of glucocorticoids and mineralocorticoids cannot be converted into androgens and might be degraded to 5α- and 3α/3β-reduced metabolites. As shown in **Figure 6B**, anti-CD3 downregulated the expression of *Srd5a1*, which encodes 5α-reductase 1, but had no significant effect on *Srd5a3*. A similar pattern of expression as that in the adrenal gland was also found in the colon, although *Srd5a1* did not reach statistical significance here (**Figure 6C**).

## Discussion

In the present study, we investigated the impact of microbiota and acute immune stress on the circulating steroid profile and expression of genes encoding local synthesis and metabolism of steroids. Given the exaggerated response of the HPA axis in the absence of microbiota (17, 18) and the impact of microbiota on neuroactive steroid levels (25), the present study was performed to determine whether microbiota modulates the steroidogenic response. Analysis was conducted in plasma and tissues collected 4 hours after injection of anti-CD3, which activates the adaptive immune system (32). We showed for the first time that there is a clear difference in the plasma steroid response to acute immune stress in GF and SPF mice, with SPF mice showing markedly different steroid patterns without corresponding changes in the expression of key enzymes of adrenal steroidogenesis.

Previous studies showed that acute psychological stress rapidly increased the plasma concentration of not only CORT but also other corticosteroids, such as PREG, PROG and DOC, and the neuroactive 5α-reduced pregnane steroids 3α,5α-THP and 3α,5α-THDOC (7, 8, 39–41), although the stress-induced increase in the plasma levels of steroids was not observed in the case of all individual steroids and all stress paradigms used. Using acute immune stimulus (physical, homeostatic stressor), we replicated the effect of acute psychological stress on plasma steroids from previous works. In SPF mice, acute immune stress increased the plasma levels of PREG, PROG, DOC, CORT, ALDO, and neuroactive steroids 3α/β,5α-THP and 3α,5α-THDOC, and some other 5α- and 20α-reduced metabolites. Our results are consistent with studies using different immune stressors, such as the endotoxin lipopolysaccharide (LPS) injection, which activates the HPA axis mainly due to the release of the cytokines IL-1, IL-6 and TNFα (42). A single injection of LPS increased plasma cortisol and 3α,5α-THP in lambs (43) and plasma CORT and ALDO in rats (44, 45). It appears that the main source of these steroids is the adrenal gland, even if the testis cannot be excluded. First, adrenalectomy inhibits the increase in plasma PROG, 3α,5α-THP and 3α,5α-THDOC levels in stressed animals, although brain levels of 3α,5α-THP were still detectable (41). Second, plasma levels of CORT and PROG are correlated and rarely dissociate during various stress responses (6), and finally, orchiectomy decreases the amount of PROG secreted during stress (7).

In contrast, the same immune stress that changes the plasma steroid levels in SPF mice had no significant effect on plasma steroids in GF animals, with the exception of CORT, which suggests that the steroid response is modulated by microbiota. The levels of corticosterone and neuroactive steroid precursors were found to be increased only in stressed SPF but not GF mice, indicating increased *de novo* steroidogenesis in SPF mice. However, plasma CORT levels were significantly increased not only in SPF but also in GF stressed mice (**Figure 2**), and the time course of the rise in plasma CORT after the application of anti-CD3 antibody did not differ between the two groups of animals (38). A potential mechanism underlying increased plasma CORT in GF mice might be linked, at least partly, to an altered regeneration of CORT. The marked increase in plasma CORT accompanied by a similar increase in 11-dehydroCORT in stressed SPF but not GF mice indicates higher systemic clearance of CORT in the presence of microbiota. The absence of upregulated 11-dehydroCORT in stressed GF mice may indicate increased conversion of 11-dehydroCORT to CORT via 11β-HSD1 and/or decreased inactivation of CORT to 11-dehydroCORT via 11β-HSD2, which is in accordance with the recently described role of peripheral antagonism of the 11β-HSD system on circulating CORT during the stress response (46). Although we did not observe any significant increase in the expression of *Hsd11b1* in the liver, colon and adrenal glands of GF mice exposed to immune stress, we cannot exclude the possibility that the reductive activity of 11β-HSD, not only in the studied tissues but also in other tissues of mice, is higher and that plasma 11-dehydroCORT of stressed GF mice is converted to CORT. It was shown that regeneration of corticosteroids from their 11-ketoderivatives by 11β-HSD in the splanchnic bed represents nearly one half of glucocorticoid synthesis in nonsplanchnic tissues (e.g., the adrenal gland) (15).

The high levels of plasma steroids in SPF mice as a response to immune challenge might also be interpreted in terms of different activities of steroidogenic and steroid metabolizing pathways in SPF and GF mice. Thus, this finding prompted us to examine the effect of acute immune stress on the transcriptional regulation of genes encoding steroid enzymes. As enzymes converting cholesterol to PREG and PROG are expressed not only in the adrenal gland but also in the testis and the enzymes converting these steroids and CORT to 3α/β-,5α- and 20α-reduced metabolites are present in many tissues, we further followed the effect of immune stress on the gene expression of these enzymes in the liver, testis, adrenal gland and colon. As reported in **Figure 5**, immune stress resulted in downregulation of genes encoding biosynthetic steroid enzymes (*Star*, *Cyp11a1*, *Hsd3b1*, *Hsd3b6*) in the testis, which was accompanied by upregulation of *Fkbp5*, a strong glucocorticoid-sensitive marker (35). These data are consistent with the known direct inhibitory effect of glucocorticoids and psychological stress on the transcription of genes encoding testosterone biosynthetic enzymes (47, 48), but do not support the possibility that the testis may participate in upregulated plasma levels of PREG and PROG in SPF mice exposed to immune stress.

The effect of immune stress on the expression of genes encoding enzymes of 3β-dehydrogenase/isomerase activity was tissue dependent. Whereas these genes were not changed (*Hsd3b2*) or downregulated in the testis (*Hsd3b1*, *Hsd3b6*), their expression was downregulated in the liver (*Hsd3b2*, *Hsd3b3*). Similarly, the expression of genes encoding 3β-HSDs, which function exclusively as 3-ketosteroid reductase (49), were downregulated in the liver (*Hsd3b4*, *Hsd3b5*) but unchanged in the testis (*Hsd3b4*). Among the genes *Akr1c6*, *Hsd17b1* and *Hsd17b2* encoding enzymes, which have the ability to catalyze multifunctional steroid activities, including 20α-HSD activity (50), we found their downregulation in the liver and the upregulation of *Hsd17b1* in the testis. From the genes encoding enzymes with 3α-HSD activity, which catalyze the transformation of 3-ketosteroids into 3α-hydroxysteroids such as 5α-DHP into 3α,5α-THP (51, 52) or the opposite reaction (53), *Dhrs9* was upregulated by anti-CD3 stimulus both in the liver and testis and *Akr1c21* in the testis, while *Akr1c14* was downregulated in the liver, similar to *Srd5a1* encoding 5α-reductase, which was downregulated both in the liver and adrenal gland. Increased expression of testicular *Akr1c21* without any decrease in *Srd5a1* and *Srd5a3* might support higher local synthesis of 3α,5α-THP and 3α,5α-DOC in anti-CD3 treated mice, an event that is not obvious in the liver, where the genes encoding 3α-reductase and 5α-reductase were downregulated. However, the effect of upregulated *Dhrs9* expression encoding 3α-HSD, which operates as an oxidase instead of reductase (53), might act in the opposite direction. Collectively, the expression of genes encoding enzymes of steroid metabolism in the testis, liver and adrenal gland was predominantly downregulated in SPF mice even if immune stress significantly upregulated their plasma steroid profile. The only gene strongly upregulated both in testis and liver was *Dhrs9*, which converts 3α,5α-tetrahydroderivatives of PROG and DOC to their 5α-dihydroderivatives (53), and thus this finding does not support the conclusion that the increased levels of 3α,5α-reduced metabolites in plasma of stressed SPF mice might be linked to upregulation of this gene. However, our data do not exclude the possibility that the observed changes in the plasma steroid profile reflect differences in the transcriptional regulation of steroid genes in other local tissues or differences in the reaction rates catalyzed by steroid enzymes in SPF and GF mice.

In conclusion, our findings underline the role of microbiota in shaping the stress response of the host. In light of evidence indicating that the gut microbiome plays in the early life a crucial role in providing essential signals for the development of the host’s immune system (54) and acute immune stress does not change the microbiota composition in gut (23), we hypothesize that the distinct impact of the acute stress response in SPF and GF mice in our study results from the absence of microbiota signals during the host’s early life. Overall, microbiota seems to alter the pathways involved in the biosynthesis, metabolism and/or clearance of steroids. In the presence of microbiota, acute immune stress increased not only the plasma level of CORT but also DOC, PREG, PROG and their metabolites, whereas in the absence of microbiota, only plasma corticosterone was significantly increased. The increased plasma levels of steroids in the presence of microbiota were not accompanied by adequate upregulated expression of genes involved in their synthesis. Our finding of stress-induced elevation of neuroactive steroids in the presence, but not in the absence, of microbiota is a valuable finding in the context of the accumulating evidence indicating the exaggerated HPA axis response to stress in GF animals (17, 18) and the role of neuroactive steroids (e.g. 3α,5α-THP, 3α,5α-THDOC) in dampening the HPA axis response to stress through effects on CRH expression (55, 56).

## Data availability statement

The raw data supporting the conclusions of this article will be made available by the authors, without undue reservation.

## Ethics statement

The animal study was approved by Animal Care Committee of the Institute of Microbiology, Czech Academy of Sciences. The study was conducted in accordance with the local legislation and institutional requirements.

## Author contributions

KV: Writing – original draft, Methodology, Project administration. TG: Funding acquisition, Methodology, Writing – original draft. MV: Conceptualization, Funding acquisition, Writing – review & editing, Methodology. PE: Methodology, Writing – review & editing. PK: Methodology, Writing – review & editing. TH: Funding acquisition, Methodology, Writing – review & editing, Supervision. DS: Methodology, Writing – review & editing. PPH: Methodology, Writing – review & editing. LN: Funding acquisition, Methodology, Writing – review & editing, Supervision. JP: Conceptualization, Funding acquisition, Writing – original draft, Methodology, Supervision.
